# Crystal Structure of the 6-Hydroxymethyl-7,8-Dihydropterin Pyrophosphokinase•Dihydropteroate Synthase Bifunctional Enzyme from *Francisella tularensis*

**DOI:** 10.1371/journal.pone.0014165

**Published:** 2010-11-30

**Authors:** Charles W. Pemble, Perdeep K. Mehta, Smriti Mehra, Zhenmei Li, Amanda Nourse, Richard E. Lee, Stephen W. White

**Affiliations:** 1 Department of Structural Biology, St. Jude Children's Research Hospital, Memphis, Tennessee, United States of America; 2 Department of Information Sciences, St. Jude Children's Research Hospital, Memphis, Tennessee, United States of America; 3 The Hartwell Center, St. Jude Children's Research Hospital, Memphis, Tennessee, United States of America; 4 Chemical Biology and Therapeutics, St. Jude Children's Research Hospital, Memphis, Tennessee, United States of America; 5 Department of Molecular Sciences, University of Tennessee Health Science Center, Memphis, Tennessee, United States of America; National Institutes of Health, United States of America

## Abstract

The 6-hydroxymethyl-7,8-dihydropterin pyrophosphokinase (HPPK) and dihydropteroate synthase (DHPS) enzymes catalyze sequential metabolic reactions in the folate biosynthetic pathway of bacteria and lower eukaryotes. Both enzymes represent validated targets for the development of novel anti-microbial therapies. We report herein that the genes which encode ^Ft^HPPK and ^Ft^DHPS from the biowarfare agent *Francisella tularensis* are fused into a single polypeptide. The potential of simultaneously targeting both modules with pterin binding inhibitors prompted us to characterize the molecular details of the multifunctional complex. Our high resolution crystallographic analyses reveal the structural organization between ^Ft^HPPK and ^Ft^DHPS which are tethered together by a short linker. Additional structural analyses of substrate complexes reveal that the active sites of each module are virtually indistinguishable from those of the monofunctional enzymes. The fused bifunctional enzyme therefore represents an excellent vehicle for finding inhibitors that engage the pterin binding pockets of both modules that have entirely different architectures. To demonstrate that this approach has the potential of producing novel two-hit inhibitors of the folate pathway, we identify and structurally characterize a fragment-like molecule that simultaneously engages both active sites. Our study provides a molecular framework to study the enzyme mechanisms of HPPK and DHPS, and to design novel and much needed therapeutic compounds to treat infectious diseases.

## Introduction

Tetrahydrofolate is an essential cofactor required for metabolic reactions involving one-carbon transfer. Most notably, it is required for the synthesis of the nucleic acid precursors purines and thymidine, the amino acids methionine and glycine, and pantothenate [1]. Higher organisms derive folate from their diet [2] and lack the necessary enzymes for folate synthesis, but almost all eubacteria and a number of lower eukaryotes including the pathogens *Plasmodium falciparum* and *Pneumocystis carinii* (*jirovecii*) synthesize tetrahydrofolate. The folate pathway is therefore an ideal target for anti-infectives. The sulfonamide drugs, which target the enzyme dihydropteroate synthase (DHPS) in the pathway, have remained important clinical agents since they were first discovered in the 1930s [3]. The folic acid pathway is also an important target for cancer therapy because the enzyme dihydrofolate reductase (DHFR) is the final enzyme in the pathway and is present in higher organisms to process dietary folate and to recycle oxidized forms of tetrahydrofolate. DHFR inhibitors such as methotrexate are potent anti-cancer agents that block nucleic acid synthesis in cancer cells [4]. Inhibiting two steps in a metabolic pathway is a particularly effective therapeutic strategy that provides a synergistic double hit, and sulfonamides in conjunction with the bacterial selective DHFR inhibitor trimethoprim have proven to be a potent and broad spectrum antibacterial cocktail that is commonly prescribed [5].

**Figure 1:**
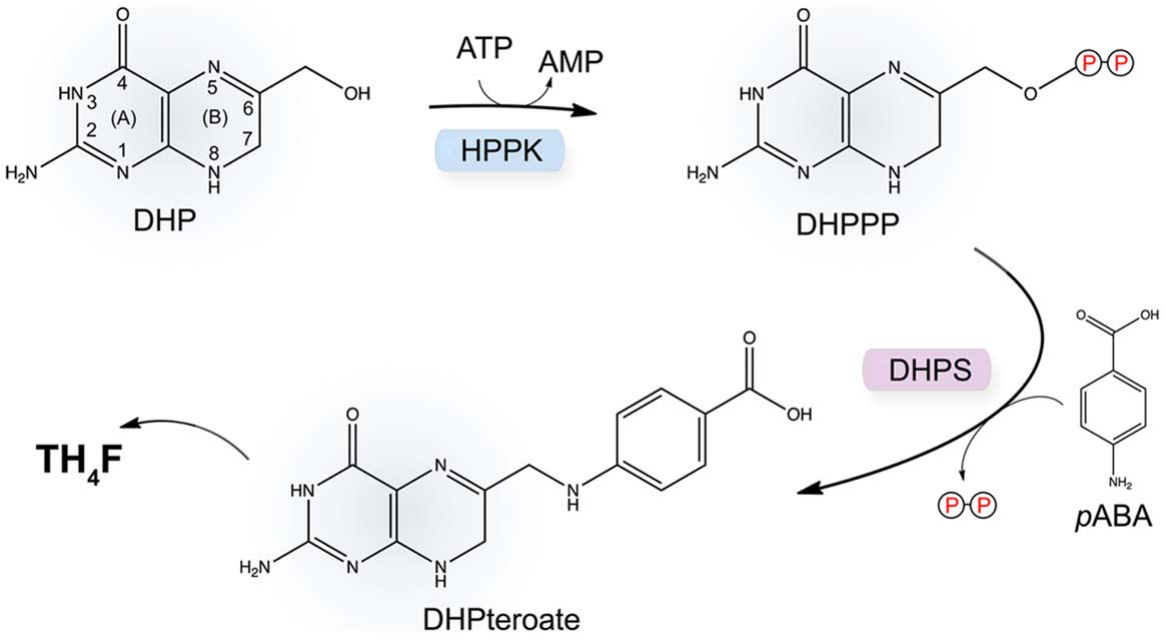
Schematic of the pyrophosphokinase (HPPK) and dihydropteroate synthase (DHPS) catalyzed reactions within the folate biosynthetic pathway. The HPPK module first uses ATP to convert 6-hydroxymethyl-7,8–dihydropterin (DHP) to 6-hydroxymethyl-7,8–dihydropterin-pyrophosphate (DHPPP) with the release of AMP, and the DHPS module then combines DHPPP with *p*-aminobenzoic acid (*p*ABA) to generate dihydropteroate (DHPteroate) with the release of pyrophosphate. The pterin-ring atoms are labeled on the DHP substrate.

DHPS acts at a crucial convergence point in the folate pathway, and catalyzes the condensation of *p*-aminobenzoic acid (*p*ABA) and 6-hydroxymethyl-7,8–dihydropterin-pyrophosphate (DHPPP) to form the intermediate dihydropteroate (Fig. 1). The sulfonamides act by mimicking *p*ABA, but their efficacy has been severely impacted by drug resistance which began to emerge shortly after they were first introduced into the clinic [6], [7], [8]. However, these orally bioavailable drugs remain useful against a number of pathogenic organisms, most notably, methicillin resistant *Stapholoccus aureus* (MRSA) and *Pneumocystis carinii* (*jirovecii*) [9], [10]. To address the problem of resistance and to continue taking advantage of this valuable broad spectrum antibacterial drug target, we are investigating a new class of DHPS inhibitors that specifically bind within the pterin-binding pocket of the active site that is structurally distinct from the *p*ABA-binding site.

Our focus is on the enzymes from three category A biowarfare agents: *Bacillus anthracis* (anthrax), *Francisella tularensis* (tularemia) and *Yersinia pestis* (plague). We described the structure of the *B. anthracis* enzyme several years ago [11] and recently reported a series of pterin-based inhibitors of the enzyme [12]. Here, we report the structure of the *F. tularensis* enzyme and show that it is fused to 6-hydroxymethyl-7,8-dihydropterin pyrophosphokinase (^Ft^HPPK) which catalyzes the previous step in the pathway (Fig. 1). This was initially revealed by searching the *F. tularensis* genome for the DHPS gene and identifying it within an open reading frame that includes the HPPK gene. The structure reveals the molecular organization of the resulting bifunctional enzyme, and we also demonstrate that each active site binds substrate in the same manner observed in the monofunctional forms. However, we also show that the distinct pterin-binding pockets of each module can each accommodate one of the pterin-based inhibitors that we have previously identified [12]. This has two important consequences for our drug discovery efforts. First, HPPK is revealed as a valid additional target for developing pterin-based folate inhibitors that can potentially block two sequential steps in the pathway. Second, the *F. tularensis* ^Ft^HPPK-DHPS bifunctional enzyme provides a convenient vehicle for identifying and developing such agents.

## Results

### Discovery of the fused gene encoding ^Ft^HPPK-DHPS

**Figure 2:**
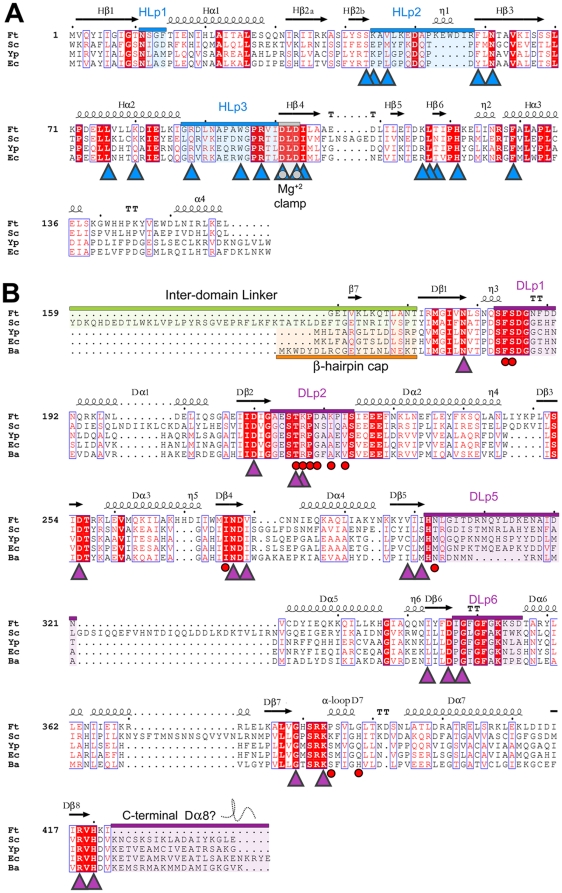
The primary structure of the HPPK-DHPS bifunctional enzyme from *Francisella tularensis* and its homology to other HPPK and DHPS enzymes. The organisms shown are *Francisella tularensis* (Ft), *Saccharomyces cerevisiae* (Sc), *Yersinia pestis* (Yp), *Escherichia coli* (Ec) and *Bacillus anthracis* (Ba), and numbering is with respect to the Ft enzyme. Secondary structure elements and key structural regions are labeled according to Fig. 3A. Strictly conserved regions are blocked in red, and conserved regions are boxed. Important loop regions are highlighted and labeled according to their domain association. (A) Multiple sequence alignment of the HPPK module. Residues that contribute to substrate binding are shown as blue triangles. The conserved motif that binds Mg^2+^ is shown as gray circles within blue triangles. (B) Alignment of the DHPS module. The inter-domain linker regions of *F. tularensis* and *S. cerevisiae* are highlighted in green and the corresponding β-hairpin of monofunctional DHPS is highlighted in orange. Residues that interact with substrates are indicated as purple triangles. Residues known to contribute to sulfonamide drug resistance are indicated by red circles. The missing Dα8 helix at the C-terminus is highlighted in purple. Sequence alignments were performed using ClustalW [39] and analyzed using ESPript2.2 [54].

When this project was initiated, the *F. tularensis LVS* genome was incomplete and unannotated, and the partially sequenced genome was received in the form of 37 contigs from the Swedish Defense Research Agency. The complete sequence has since been published [13]. The sequences were analyzed using a combination of programs within the GCG software suite (Accelrys Software Inc. San Diego, CA), EMBOSS (The Sanger Center, Hinxton, UK), and NCBI (Bethesda, MD). Synteny was identified through alignment with the DHPS enzyme from *B. anthracis* which revealed the *F. tularensis* DHPS gene on the antisense strand. Analysis of the open reading frame revealed that the ^Ft^DHPS gene is considerably longer than the typical prokaryotic DHPS gene, and that a 5′ extension encodes the ^Ft^HPPK gene. Multiple alignments showed that the two sequences are well conserved compared to those of the monofunctional enzymes, particularly in the regions of the active and substrate-binding sites (Fig. 2). However, the C-terminal residues of the ^Ft^DHPS module corresponding to the final α-helix of the TIM-barrel structure is missing, and it was of particular interest to understand how the structure would accommodate this missing α-helix and whether it had any functional consequences.

### ^Ft^HPPK-DHPS Apo Structure

**Figure 3:**
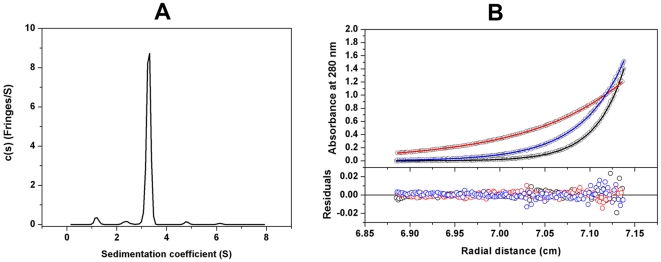
Analytical ultracentrifugation of the HPPK-DHPS bifunctional enzyme from *Francisella tularensis*. (A) The sedimentation velocity profiles (fringe displacement) were fitted to a continuous sedimentation coefficient distribution model c(s). The experiment was conducted at a loading protein concentration of 0.69 mg/ml in at 20°C and at a rotor speed of 60,000 rpm. (B) Absorbance scans at 280 nm at equilibrium are plotted *versus* the distance from the axis of rotation. The protein was centrifuged at 4°C for at least 24 h at each rotor speed of 15 k (red), 22 k (blue) and 27 k (black) rpm. The *solid lines* represent the global nonlinear least squares best-fit of all the data sets to a monomer-dimer self-association model with a very weak K_D_ (2.7 mM). The loading protein concentration was 20 µM and the r.m.s. deviation for this fit was 0.0037 absorbance units.

^Ft^HPPK-DHPS crystallized in space group P1 with two molecules in the unit cell, and the structure was determined to 2.2 Å using molecular replacement methods (Table 1; Protein Data Bank accession code 3MCM). Size-exclusion chromatography suggested that the enzyme is a monomer in solution (data not shown) and that the crystallographic dimer is unlikely to have any functional significance. To confirm this, we further characterized the protein in solution by analytical ultracentrifugation, specifically using sedimentation velocity and equilibrium analysis assays. Both experiments showed that ^Ft^HPPK-DHPS exists mainly as a monomer in solution (Fig. 3), and there are no dimers observed in the c(s) distribution profile (Fig. 3a) at the concentration used. The standard sedimentation coefficient (*s_20,w_*) obtained from the analysis (3.41S) corresponds to a molar mass of 53,000 Da, close to the theoretical monomer molecular mass of 50,509 Da for this protein. The weight-average frictional ratio value (*f/f_0_*)_w_ obtained from the analysis (1.39) reflects a slightly elongated globular protein, consistent with the three-dimensional structure described below. It was possible to detect a weak monomer-dimer association in solution that may explain the dimer observed in the crystal structure. The dissociation equilibrium constant (K_D_) of the monomer-dimer self-association model determined from the equilibrium data was 2.7 mM (Fig. 3b).

**Table 1:** Data Collection and Refinement Statistics.

	Apoenzyme	Substrate Complex	Compound 1 Complex
**Data collection**			
Space group	P1	P1	P1
Cell dimensions			
a, b, c (Å)	43.0, 58.1, 105.7	42.5, 58.5, 109.3	42.9, 58.2, 105.1
α, β, γ (°)	91.3, 99.3, 111.6	82.0, 81.0, 68.1	91.0, 80.1, 68.3
Resolution (Å)	38.6−2.2 (2.28−2.2)*	39.2−2.3 (2.38−2.3)*	46.1−2.2 (2.28−2.2)*
R_merge_	0.13 (0.28)	0.13 (0.30)	0.11 (0.33)
Completeness (%)	94.4 (80.1)	96.3 (84.9)	93.3 (76.4)
Redundancy	3.5 (2.9)	3.5 (2.8)	3.6 (2.8)
I/σI	23.6 (5.3)	24.8 (4.4)	21.7 (4.6)
**Refinement**			
Total reflections	155,578	143,185	157,212
Unique reflectionsResolution (Å)	44,08338.6−2.2	41,09939.2−2.3	43,51946.1−2.2
R_work_/R_free_(%)^a^	20.6/25.6	26.2/30.8	21.3/25.9
No. of atomsProteinWaterMgAMPcPPDHPCompound 1Average B-factor (Å^2^)	6,0431021–––	6,3274246256–	5,9761203––22
Protein	47.8	43.7	46.6
Water	35.8	31.5	35.2
Mg^2+^	60.2	30.8	51.5
AMPcPP	–	27.3	–
DHP	–	27.6	–
Compound 1	–	–	53.0
Ramachandran (%)			
Favored	97.4	96.2	96.2
Allowed	2.6	3.8	3.8
Outliers	0	0	0
Rmsd			
Bond lengths (Å)	0.01	0.01	0.01
Bond angles (°)	0.9	0.96	0.74

*Data were collected from a single crystal. Values in parentheses are for the highest-resolution shell.

a R_free_ was calculated using 5% of the reflections.

**Figure 4:**
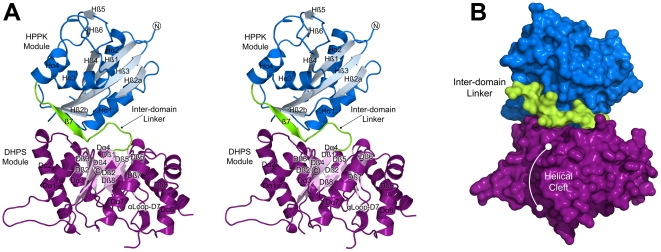
The overall structure of the HPPK-DHPS bifunctional enzyme from *Francisella tularensis*. (A) A stereo view of the overall fold and domain organization showing the secondary structure elements within each module. Each element is labeled with the prefixes ‘H’ and ‘D’ to reflect their locations in the HPPK (blue) and DHPS (purple) domains, respectively. The N- and C-termini and the linker region (green) are labeled. Note that helix Dα8 in the canonical DHPS TIM-barrel is missing. (B) A surface representation of the view shown in (A) that highlights the position of the domain linker and the cleft within the DHPS module corresponding to the missing Dα8 TIM-barrel α-helix.

An overview of the structure is shown in Figure 4A. The N-terminal ^Ft^HPPK module is connected to the C-terminal ^Ft^DHPS module by a structured linker region, and the structures of each module are very similar to those of the monofunctional enzymes. A multiple sequence alignment using representative HPPK and DHPS primary structures confirms the high degree of sequence conservation within each module (Fig. 2). The two molecules in the asymmetric unit are very similar (RMSD of 0.4 Å on α-carbons) and differ only in the flexible loop regions. Regions missing in the final model due to disorder are residues 44–56 and 89–98 within the ^Ft^HPPK module, and residues 213–224, 304–318 and 354 within the ^Ft^DHPS. In addition, there was no observable electron density for the N-terminal 20 residues that correspond to the His_6_-tag. In the descriptions of the modules, secondary structures are numbered according to the monofunctional enzymes with prefixes H and D for HPPK and DHPS, respectively (Fig. 2).

The core of the ^Ft^HPPK module adopts the canonical αβα fold that has previously been described [14], [15], [16] and comprises a central, 4-stranded antiparallel β-sheet (Hβ2-Hβ3-Hβ1-Hβ4) flanked by four α-helices (Hα1-Hα2-Hα3-Hα4). Helices Hα1 and Hα2 pack against the surface of one side of the β-sheet, and Hα3 and Hα4 pack onto the other surface. The C-termini of related monofunctional HPPKs typically end following Hα4 but the ^Ft^HPPK module terminates in a well-ordered 10-residue inter-domain linker that directly tethers it to ^Ft^DHPS. The linker contains a short, 4-residue β-strand that associates with the β-sheet of ^Ft^HPPK. The TIM-barrel fold of the ^Ft^DHPS module [11], [17], [18], [19] contains the typical 8-stranded β-barrel (Dβ1-Dβ2-Dβ3-Dβ4-Dβ5-Dβ6-Dβ7-Dβ8). However, as anticipated from the sequence alignment (Fig. 2), only seven of the eight surrounding α-helices are present (Dα1-Dα2-Dα3-Dα4-Dα5-Dα6-Dα7) with the carboxy-terminal Dα8 helix missing. The gap in the TIM barrel is partly filled by Lys421 and Ile422 that follow Dβ8 at the C-terminus, and by the flanking helices Dα1 and Dα7 that move slightly inwards to fill the space, but there remains a significant cleft on the surface of the TIM-barrel structure (Fig. 4B). In the *E. coli* and *B. anthracis* DHPS structures, there is an N-terminal β-hairpin that caps the N-terminal end of the TIM-barrel, but this is not present in the *F. tularensis* structure. The ^Ft^HPPK domain partially performs this role, but is shifted by approximately 30° from where the β-hairpin would typically be positioned.

### ^Ft^HPPK-DHPS in Complex with HPPK Substrates

**Figure 5:**
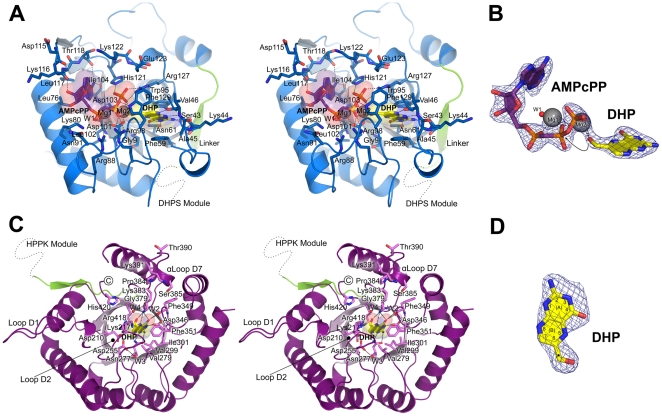
The ^Ft^HPPK and ^Ft^DHPS modules bound to substrates. (A) Stereo view of the ^Ft^HPPK module showing the detailed interactions with AMPcPP and DHP. Both substrates are covered with transparent molecular surfaces and gray dashed lines indicate putative hydrogen-bond interactions. Residues that contribute to substrate binding are labeled and shown in blue sticks. The inter-domain linker is colored green and a dashed line indicates the position of the carboxy-terminal ^Ft^DHPS module. (B) Electron densities for the nucleotide analog AMPcPP (purple) and DHP (yellow) bound to the HPPK module. Two Mg^2+^ ions (gray spheres labeled Mg1 and Mg2), and an active site water (red sphere labeled W1) are also shown. The arrow indicates how the 6-hydroxymethyl group of DHP is appropriately oriented towards the pyrophosphate moiety of AMPcPP for in-line phosphoryl transfer. (C) Stereo view of the interactions between DHP and the ^Ft^DHPS module. DHP is bound within the TIM-barrel (light pink, β-barrel), and the residues that mediate the interaction are labeled and shown in pink sticks. Three structural water molecules are shown as red spheres and are labeled W2, W3 and W4. The location of the ^Ft^HPPK module is indicated by a dashed line that extends from the inter-domain linker (green). (D) Electron density for the molecule of DHP (yellow) which bound in the pterin pocket of the ^Ft^DHPS module. In (B) and (D), the *F*o-*F*c simulated-annealing omit electron densities are contoured at 3.5 σ.

The crystal structure of ^Ft^HPPK-DHPS in complex with the non-hydrolyzable ATP analog (AMPcPP) and 6-hydroxymethyl-7,8-dihydropterin (DHP) was determined at 2.3 Å resolution (Table 1; Protein Data Bank accession code 3MCO). An α-carbon superposition shows that the overall fold of the substrate complex closely resembles that of the apo form with an RMSD of 0.6 Å. The mode of interaction of the two substrates within the ^Ft^HPPK active site (Fig. 5A) is virtually identical to that of previously determined HPPK complexes [15], [16], [20], [21], [22]. Difference electron density maps clearly showed both substrates bound to the ^Ft^HPPK module, and two large spheres of density within the ATP binding pocket were interpreted and successfully refined as Mg^2+^ ions (Fig. 5B). The HPPK mechanism involves three flexible loops, HLp1, HLp2 and HLp3 in our structure, that undergo large conformational changes and adopt more stable structures in the presence of the two substrates [20], [23], [24], [25]. Lys44, Ala45 and Val46 within HLp2 and Arg88, Trp95 and Arg98 within HLp3 make key stabilizing interactions with the two substrates.

The adenosine ring of AMPcPP packs into a cleft formed by Leu76, Lys80, Ile104, Leu117, Thr118 and His121, and the triphosphate moiety is coordinated by the two Mg^2+^ ions bridged between two absolutely conserved aspartate residues, Asp101 and Asp103. In addition, there are key electrostatic interactions between the β- and γ-phosphates and Lys80, Arg88, Arg98, His121, and Arg127 that are also highly conserved. In one of the molecules of the asymmetric unit in the apo structure, one Mg^2+^ ion remains coordinated between the side chains of Asp101 and Asp103 in the absence of substrate, but the relatively high B factors of the ion and its coordinating oxygen atoms suggest that it is weakly bound. The ribose moiety mostly points towards solvent, although the 2′-hydroxyl is hydrogen bonded to the main-chain oxygen atom of Lys116. The DHP substrate binds within an adjacent pocket formed by Ser43, Val46, Asn61 and Trp95, and the pterin ring π-stacks between two highly conserved aromatic residues, Phe59 and Phe129. The OG oxygen of Ser43, together with the mainchain of residues 44 and 46, provide a hydrogen bond ‘zipper’ that specifically recognizes the ‘nitrogen face’ of the pterin ring (positions 1, 2, and 8 as defined in Fig. 1), and the side chain of Asn61 forms hydrogen bonds to the nitrogen and carbonyl-oxygen substituents at positions 3 and 4. Within this pocket, the 6-hydroxymethyl group of DHP is coordinated by one of the Mg^2+^ ions and appropriately oriented towards the pyrophosphate moiety of AMPcPP for in-line phosphoryl transfer (Fig. 5B).

We recently showed that DHP can engage the pterin-binding pocket of the monofunctional *B. anthracis* enzyme and act as an effective inhibitor [12]. It was therefore not surprising that a second molecule of DHP was present in the pterin-binding pocket of the DHPS module (Fig. 5C). The electron density for this second DHP is unequivocal (Fig. 5D). We have extensively characterized the pterin-binding pocket of DHPS and described how the pterin ring is recognized [11], [12], [26], and the key residues are conserved in the *F. tularensis* enzyme. These residues include Asp210, Thr216, Asp255, Asn277, Val279, Val299, Ile301, Asp346, Phe349, Phe351, Gly379, Lys383 and Arg418, within the core of the TIM-barrel, which provide specific van der Waals, hydrogen-bond and π-stacking interactions. A key structural water molecule (W2) is also present.

### The Structures of the Active Site Loops in the DHPS Module

Loops D1 and D2 in the ^Ft^DHPS module that link the first two αβ units of the TIM barrel are highly conserved, contain sites of sulfonamide resistance (Fig. 2) and clearly have important but poorly defined functional roles [11]. In both molecules in the asymmetric unit, D1 (residues 180 to 193) is folded into an extended β-ribbon and makes a crystal contact with a neighboring molecule via Phe189 (Fig. 5C). A similar conformation is present in our *B. anthracis* DHPS structure which precludes a prediction of its role at the active site, although a conserved aspartic acid that has been implicated in catalysis [19] is present (Asp186). In contrast, D2 is relatively well ordered adjacent to the active site, and completely visible in molecule ‘B’ in the presence of DHP substrate (Fig. 5C). Lys217, typically an arginine residue in other DHPS enzymes, can potentially interact with the carboxyl group of *p*ABA or contribute to the anion-binding pocket that engages the β-phosphate of the DHPPP substrate. This location for D2 adjacent the *p*ABA binding site is consistent with the presence of sulfonamide resistance mutations within this loop region. Finally, in other DHPS structures, the loop connecting Dβ7 and Dα7 typically contains a short two-turn α-helix, α-Loop D7, that we have shown interacts with the carboxyl group of *p*ABA in our *B. anthracis* structure bound to pteroic acid [11]. This helix is limited to a single turn in the *F. tularensis* DHPS module and terminated by Pro384, but Lys383 and Ser385 remain well positioned to stabilize the binding of the *p*ABA moiety like their counterparts in *B. anthracis*.

### ^Ft^HPPK-DHPS bound to a DHPS inhibitor

**Figure 6:**
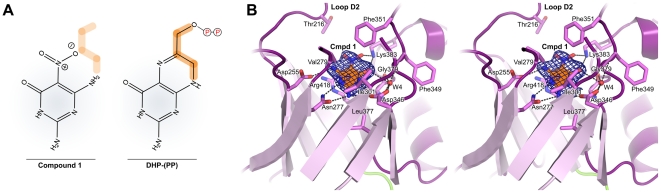
Interaction of the ^Ft^DHPS module with Compound 1. (A) Schematic comparison between the scaffolds of Compound 1 and DHP-PP. Compound 1 comprises a pterin-like core and is missing half of the B-ring as highlighted in orange. (B) Stereo view of Compound 1 (orange) bound within the pterin pocket of the TIM-barrel. Residues that make van der Waals and hydrogen-bond contacts are labeled and shown as pink sticks. The *F*o-*F*c simulated-annealing omit electron density for Compound 1 is shown as a blue mesh contoured at 3.5 σ.

We recently reported a series of DHPS inhibitors that target the pterin-binding pocket using a virtual screening approach based on our structural studies of the *B. anthracis* enzyme [12], [26]. The goal of these studies is to develop new antibacterial compounds that bypass the problems of resistance associated with the sulfonamides which target the *p*ABA binding site of the enzyme. One of the compounds, *2-(7-amino-1-methyl-4,5-dioxo-1,4,5,6-tetrahydorpyrimido[4,5-c]pyridazin-3-yl)propanoic acid* (Compound 1, Fig. 6A), is a low molecular weight fragment-like molecule that is well suited for elaboration and further development, and it was of interest to evaluate its binding within the *F. tularensis* enzyme. The crystal structure of ^Ft^HPPK-DHPS in complex with Compound 1 was successfully resolved and refined to 2.2 Å resolution (Table 1; Protein Data Bank accession code 3MCN). The small molecule binds within the pterin pocket in exactly the same way as the *B. anthracis* enzyme (Fig. 6B), engaging the pterin recognition residues Asp255, Asn277, Asp346, and Lys383 and a structural water molecule in hydrogen bonding and electrostatic interactions, and the guanidinium moiety of Arg418 in a π-stacking interaction.

**Figure 7:**
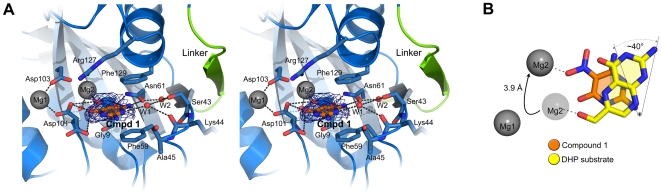
The ^Ft^HPPK module bound to Compound 1. (A) Stereo view showing the interactions of Compound 1 (Cmpd 1) within the DHP binding pocket. The orientation is the same as that shown in Fig. 5A. Putative hydrogen-bonds are indicated as gray dashes. Two water molecules (W1 and W2) are shown as red spheres bridging between Compound 1 and loop H2. Note that the side chain of Asp101 is 50∶50 in two orientations, both of which engage Compound 1. Compound 1 is enclosed by the *F*o-*F*c simulated-annealing omit electron density contoured at 2.5 σ (grey mesh) and 5.0 σ (royal blue). The latter indicates the most probable location of the electron-rich nitro-moiety which dictated the fit. (B) Comparison between the binding orientations of DHP (yellow) and Compound 1. As measured with respect to their ‘nitrogen faces’, the two compounds are rotated by ∼40°. The magnesium ions in the substrate complex at the first and second positions are labeled Mg1 and Mg2, respectively, and interaction with Compound 1 causes Mg2 to bind in a new location as indicated by the arrow.

Intriguingly, Compound 1 was also found within the pterin pocket of the ^Ft^HPPK module (Fig. 7A). Similar to the HPPK substrate and consistent with its pterin-like structure, the molecule stacks between the conserved phenylalanine side-chains (Phe59 and Phe129) and forms a hydrogen bonding interaction with Asn61. However, comparing the ‘nitrogen faces’ of Compound 1 and DHP that dictate their similar binding to the DHP pocket, a rotation of ∼40° is revealed which orients the nitro moiety proximal to the Mg^2+^ ion within the divalent metal binding site (Fig. 7B). To accommodate the molecule, the Mg^2+^ ion is shifted nearly 4 Å, presumably to coordinate the partial negative charge associated with the nitro group. A consequence of this rotation is that the ‘nitrogen face’ does not directly engage residues 43–47 but instead interacts with loop H2 via a water molecule that occupies this void. Also, the side chain of Asp101 can engage Compound 1 via two equally populated orientations. Finally, in the absence of AMPcPP, residues 89–96 in loop H3 remain disordered, and Trp95 does not enclose the pterin pocket. Thus, while Compound 1 closely mimics the binding of substrate in the ^Ft^DHPS module, it only partially mimics substrate binding in the ^Ft^HPPK module.

## Discussion

### Comparison with monofunctional HPPK and DHPS enzymes

To date, the structures of HPPK and DHPS from eubacteria have revealed monofunctional enzymes, and our discovery that the two activities are fused into a single polypeptide in *F. tularensis* is therefore somewhat surprising. Nevertheless, a comparison of the fused enzyme with its monofunctional counterparts reveals close similarities and common active sites. The structure of HPPK has been determined to very high resolution in its substrate-bound form [21], and the catalytic mechanism is well understood and supported by extensive kinetic and mutagenesis data [22], [27], [28], [29], [30]. The canonical αβα fold of the ^Ft^HPPK module is particularly well conserved and incorporates three conserved loop regions, HLp1, HLp2 and HLp3, which are centrally involved in substrate binding (Fig. 5A). In the substrate-bound state, where these active site loops are well structured, the ^Ft^HPPK core can be superimposed on the *E. coli* and *Y. pestis* enzymes with <1.5 Å RMSD. A total of 13 residues were identified as being absolutely conserved for structural and catalytic reasons [20] and two of these are aromatic residues that clamp the pterin ring of the DHP substrate. HLp2 is slightly larger in ^Ft^HPPK and locally organized by Trp55 that spatially replaces Gln50 of the *E. coli* enzyme. Similar to other HPPKs, the three loops help to stabilize the interaction of substrates via an intricate hydrogen-bonding network, and two essential catalytic Mg^2+^ ions coordinate the phosphate groups of ATP and the nucleophilic hydroxyl group of DHP. The most significant difference in the ^Ft^HPPK module occurs at the C-terminus that contains the linker region to the ^Ft^DHPS module.

The TIM-barrel fold of the ^Ft^DHPS module is also structurally well conserved, and can be superimposed (α-carbon) on our *B. anthracis* DHPS structure with an RMSD of 1.9 Å. Although the DHPS catalytic mechanism has yet to be fully elucidated, the key catalytic and substrate binding residues that have been identified [11] are all present. These include residues that define the pterin-binding pocket and others within the two flexible loops DLp1 and DLp2 that are proposed to close over the active site locale [11]. However, there are several significant differences. Most notable is the absence of the eighth TIM-barrel α-helix located at the C-terminus. This is curious because the deletion creates a cleft in the ^Ft^DHPS module that would be predicted to destabilize the TIM-barrel. Functionally, the missing α-helix is unimportant, but it is part of the dimer interface of the monofunctional counterparts [11]. We have unequivocally shown that the bifunctional ^Ft^HPPK-DHPS enzyme is monomeric, and the deletion of the α-helix may therefore preclude dimer formation. The ^Ft^DHPS module also lacks an N-terminal β-ribbon that would block the interface with ^Ft^HPPK, and its absence is apparently structural. Finally, the non-TIM-barrel α-helix, α-loop D7, that points toward the active site is much shorter in the ^Ft^DHPS module, although a serine and lysine residue that are required to interact with *p*ABA and pterin substrates are present [11].

### A Putative Alternate Conformation

The fusion of metabolic pathway enzymes is commonly observed in higher organisms and is exemplified by the fatty acid synthase complex [31], [32] that essentially comprises fused eubacterial enzymes [33]. Although absent in higher eukaryotes, the folate pathway is present in some lower eukaryotes where the enzymes can be fused, as exemplified by the trifunctional DHNA-HPPK-DHPS enzymes that have been characterized in many fungi. The structure of the ^Sc^HPPK-DHPS region from *Saccharomyces cerevisiae* has been determined [34], and although it is similar to our ^Ft^HPPK-DHPS structure, the linker region is much longer (50 residues versus 13 residues) and the HPPK modules are rotated with respect to the DHPS module by some 60°. The fusion of modules presumably increases pathway efficiency, either by locally concentrating sequential intermediates or, as exemplified by the fatty acid synthase complex, directly channeling intermediates from one active site to the next. In both the *F. tularensis* and *S. cerevisiae* HPPK-DHPS structures, the active sites are on opposite sides of the fused molecule and not appropriately positioned to channel substrates. This observation is consistent with kinetic data from a plant mitochondrial HPPK/DHPS fused enzyme which suggest that substrate channeling does not occur although the individual reactions are coupled [35].

However, our structure does suggest an alternate and more stable conformation, consistent with the analytical ultracentrifugation data, in which the linker region engages the cleft on the ^Ft^DHPS module generated by the missing α-helix. If this is the case, why are both molecules in the asymmetric unit in the same extended conformation? Inspection of the crystal packing reveals that the extended DLp1 loops of both ^Ft^DHPS modules in the asymmetric unit interact with the inter-module interfaces of their neighbors in a very similar fashion, essentially stabilizing the extended conformation. Without this crystal contact, the ^Ft^HPPK-DHPS interface is relatively small (∼485 Å^2^ as determined by AREAIMOL [36]). We intend to truncate loop DLp1 to investigate new crystal forms of the enzyme that lack this crystal contact and which might allow this putative alternate conformation to be visualized.

### Pterin Pocket Inhibitors

We have recently demonstrated that the pterin pocket of DHPS can bind an array of pterin-like molecules and represents an attractive target for anti-folate drug discovery [12]. We have observed the HPPK substrate DHP in the pterin pocket of the *B. anthracis* enzyme and measured the IC_50_ at 58.4 µM [12], and our observation that DHP can also bind within the pterin pocket of ^Ft^DHPS is therefore not surprising. Using a linked assay in which the active ^Ft^HPPK module generates the unstable DHPS substrate DHPPP from DHP, we have not been able to measure the ^Ft^DHPS activity, and this is probably a result of the inhibition by excess DHP. We have no reason to believe that the ^Ft^DHPS module is non-functional. The active and substrate-binding sites are both intact and there is no other *folP* gene encoding a second DHPS isozyme in the organism. Furthermore, a primary structure alignment of ^Ft^HPPK-DHPS from various *F. tularensis* strains reveals a wild type sequence, and no detrimental mutations have been acquired in the attenuated *LVS* strain we are using.

In contrast, the presence of the DHPS inhibitor in the pterin pocket of ^Ft^HPPK is very surprising and has important implications for drug discovery. The inhibitor was specifically identified using the DHPS pterin pocket as the target and it was not anticipated to engage the HPPK pocket which has a very different architecture. HPPK has long been recognized as a potentially useful target for the development of new antibacterials which prompted the original crystallographic analyses [14], [15], [16], and we will now pursue this possibility using our panel of DHPS pterin-like inhibitors [12]. Moreover, the *F. tularensis* enzyme and its structure will facilitate studies to identify molecules that simultaneously bind to both pockets. Antifolate cocktails such as sulfamethoxazole/trimethoprim that inhibit DHPS and DHFR in the folate pathway are potent and widely-used antibacterial agents. It has been noted that a cocktail or a single agent that inhibits the HPPK and DHPS activities could be similarly efficacious [15]. HPPK and DHPS use ordered enzyme mechanisms in which the ATP cleft of the former or the pterin pocket of the latter is first occupied, followed by the binding of DHP or *p*ABA, respectively, and both enzymes use loop conformational changes to assemble the active site. We have shown that the flexible loops and pABA are not required for the binding of pterin pocket inhibitors in DHPS [12] and we now show that the same is true for HPPK with respect to its flexible loops and ATP. This will facilitate future drug discovery efforts with this enzyme.

## Materials and Methods

### Ethics Statement

N/A

### Protein expression and purification

The 50 kDa, ^Ft^HPPK-DHPS enzyme from *F. tularensis LVS* was cloned and expressed using the pET28a vector containing an N-terminal His_6_-tag, grown at 37°C in BL21 *Escherichia coli* cells, induced with 0.5 mM IPTG at 18°C and harvested after 4 hours. Cells were lysed and centrifuged, and the supernatant containing soluble His_6_-tagged protein was passed over a nickel-nitrilotriacetic acid (NTA) HisTrap HP affinity column (GE Healthcare). Following elution using a linear gradient of 20 mM Tris (pH 7.6), 500 mM NaCl, and 500 mM imidazole, relevant fractions were verified by SDS-PAGE, pooled, and treated with 2 mM EDTA and 15 mM dithiothreitol (DTT). For crystallization purposes, the His_6_-tag was left fused to ^Ft^HPPK-DHPS. Fractions were concentrated, filtered (0.45 µm), and further purified to homogeneity using a Superdex 75 size exclusion column (GE Healthcare) equilibrated with 20 mM HEPES (pH 7.5), 100 mM NaCl, 1 mM EDTA and 0.5 mM DTT. Purified ^Ft^HPPK-DHPS was concentrated to 26 mg/ml, filtered (0.22 µm), aliquoted, flash frozen in liquid nitrogen and stored at −80°C.

### Crystallization and structure determination

Crystals of the apo-enzyme form of ^Ft^HPPK-DHPS were grown using the sitting-drop vapor diffusion method. Prior to crystallization, ^Ft^HPPK-DHPS was diluted to 10 mg/ml in a solution that contained 20 mM HEPES (pH 7.5), 100 mM NaCl, 1 mM EDTA, 1 mM DTT and 50 mM MgCl_2_. Initial crystals were found by screening against the JCSG Core I-IV suites (Qiagen) using a Phoenix robot system (Art Robbins Instruments). Further optimization yielded diffraction quality crystals and were obtained by mixing equal volumes of the ^Ft^HPPK-DHPS mixture and a precipitant solution containing 90 mM Tris (pH 8.0), 190 mM Sodium acetate, 24% (w/v) polyethylene glycol (PEG) 4000, 17% glycerol, and allowed to equilibrate at 18°C. Crystals appeared in approximately 3 days and reached maximum size in about 1 week. The presence of 17% glycerol in the reservoir solution served as a cryo-protectant, and crystals were frozen by direct immersion in liquid nitrogen. Substrate (1) and inhibitor (2) complexes were obtained by soaking pre-grown crystals in (1) excess 6-hydoxymethyl-7,8-dihydropterin powder (DHP, Schircks Laboratories) and 50 mM α,β-methyleneadenosine 5′-triphosphate (AMPcPP, Sigma), and (2) *2-(7-amino-1-methyl-4,5-dioxo-1,4,5,6-tetrahydorpyrimido[4,5-c]pyridazin-3-yl)propanoic acid* (Compound 1) as a crystalline powder due to solubility problems.

Diffraction data were collected on the SER-CAT beamline 22-ID at the Advanced Photon Source (APS) at Argonne National Laboratory, and processed using HKL2000 [37] (Table 1). The apo-structure was solved by maximum-likelihood molecular replacement (MR) using the program *Phaser* [38], and the coordinates of HPPK from *Y. pestis* (PDB ID 2QX0) and DHPS from *B. anthracis* (PDB ID 1TWW) were used as search models. Significant editing of both models was required. The alignment of sequences was performed using ClustalW [39], and the program CHAINSAW [40] was used to prune non-conserved residues to the last common atom. Homology modeling using the SWISS-MODEL server [41] coupled with alignments of known structures guided the strategic removal of flexible loops and helped define a reasonable subdomain division between the ^Ft^HPPK and ^Ft^DHPS modules. The bifunctional enzyme was divided into its individual ^Ft^HPPK and ^Ft^DHPS subdomains, searching first for the ^Ft^DHPS module, and the appropriate positioning of the two modules relative to the intra-domain linkage validated our eventual MR solution. Iterative structure refinement was carried out using a combination of CNS1.2 [42] and REFMAC5 [43] for simulated annealing, sigma A weighted composite omit electron density calculations, and restrained maximum likelihood refinement. The initial model was manually rebuilt using COOT [44]. The final models of the apo-enzyme form, the ternary complex bound to AMPcPP and DHP substrates, and ^Ft^HPPK-DHPS inhibited by Compound 1 were fully refined using restrained options within REFMAC5 and the PHENIX software suite [45]. The data statistics for refinement are summarized in Table 1. The molecular coordinates and topologies of the ligands AMPcPP, DHP, and Compound 1 were generated using either the HIcUP or PRODRG2 [46] servers. The quality of each crystal structure was determined using MOLPROBITY [47], and the Ramachandran statistics are reported in Table 1. AREAIMOL [36] was used to identify surface residues that interact with substrate and inhibitor through either hydrophobic or polar contacts. Other electrostatic and surface calculations were performed using APBS Tools [48] and CASTp [49], respectively.

### Analytical Ultracentrifugation

Experiments were carried out in a ProteomeLab XL-I analytical ultracentrifuge with a four-hole rotor (Beckman An-60Ti) and cells containing sapphire or quartz windows and charcoal-filled Epon double-sector centre pieces (Beckman Coulter, Fullerton, CA). The density and viscosity of the ultracentrifugation buffer, 20 mM HEPES pH 7.5, 100 mM NaCl, 1 mM EDTA and 1 mM DTT at 4 and 20°C were calculated from its composition. The partial specific volume at 4 and 20°C and the molecular weight of the protein was calculated based on its amino acid composition using the software SEDNTERP [50]. All samples were dialysed against the ultracentrifugation buffer and the dialysate was used as an optical reference. For the sedimentation velocity experiment the loading volume of 400 µl was identical for the reference and sample chambers of the double-sector centrepiece. Fringe displacement data at time intervals of 1.0 min were collected with the Rayleigh interference system for 10 hours at a rotor speed of 60,000 rpm and analysed with SEDFIT software (www.analyticalultracentrifugation.com) using the model for continuous sedimentation coefficient distribution *c(s)* with deconvolution of diffusional effects [51], [52]. The sedimentation coefficient distribution *c(s)* was calculated with maximum entropy regularization at a confidence level of p = 0.68 and at a resolution of sedimentation coefficients of n = 100. The positions of the meniscus and bottom, as well as time-invariant and radial noises, were fitted. Sedimentation equilibrium was attained at 24 h at a rotor temperature of 4°C at increasing speeds of 15, 22 & 27 k rpm [53]. Protein at a concentration of 20 µM (120 µL) was loaded into double-sector centrepieces and absorbance distributions recorded at 280 nm in 0.001 cm radial intervals with 20 replicates for each point. Global least squares modelling were performed at multiple rotor speeds with the software SEDPHAT (www.analyticalultracentrifugation.com) using a reversible monomer-dimer self-association model as well as the single species model [53].
